# Weighted-persistent-homology-based machine learning for RNA flexibility analysis

**DOI:** 10.1371/journal.pone.0237747

**Published:** 2020-08-21

**Authors:** Chi Seng Pun, Brandon Yung Sin Yong, Kelin Xia

**Affiliations:** 1 Division of Mathematical Sciences, School of Physical and Mathematical Sciences, Nanyang Technological University, Singapore, Singapore; 2 School of Biological Sciences, Nanyang Technological University, Singapore, Singapore; Beijing University of Posts and Telecommunications, CHINA

## Abstract

With the great significance of biomolecular flexibility in biomolecular dynamics and functional analysis, various experimental and theoretical models are developed. Experimentally, Debye-Waller factor, also known as B-factor, measures atomic mean-square displacement and is usually considered as an important measurement for flexibility. Theoretically, elastic network models, Gaussian network model, flexibility-rigidity model, and other computational models have been proposed for flexibility analysis by shedding light on the biomolecular inner topological structures. Recently, a topology-based machine learning model has been proposed. By using the features from persistent homology, this model achieves a remarkable high Pearson correlation coefficient (PCC) in protein B-factor prediction. Motivated by its success, we propose weighted-persistent-homology (WPH)-based machine learning (WPHML) models for RNA flexibility analysis. Our WPH is a newly-proposed model, which incorporate physical, chemical and biological information into topological measurements using a weight function. In particular, we use local persistent homology (LPH) to focus on the topological information of local regions. Our WPHML model is validated on a well-established RNA dataset, and numerical experiments show that our model can achieve a PCC of up to 0.5822. The comparison with the previous sequence-information-based learning models shows that a consistent improvement in performance by at least 10% is achieved in our current model.

## 1 Introduction

Biomolecular functions usually can be analyzed by their structural properties through quantitative structure-property relationship (QSPR) models (or quantitative structure-activity relationship (QSAR) models). Among all the structural properties, biomolecular flexibility is of unique importance, as it can be directly or indirectly measured by experimental tools. Debye-Waller factor or B-factor, which is the atomic mean-square displacement, provides a quantitative characterization of the flexibility and rigidity of biomolecular structures. With the strong relationship between structure flexibility and functions, various theoretical and computational methods have been proposed to model the flexibility of a biomolecular. Such methods include molecular dynamics (MD) [1], normal mode analysis (NMA) [2–5], graph theory [6], elastic network models (ENMs) [7–12], Gaussian network model (GNM) [7, 8], anisotropic network model (ANM) [9], local density model (LDM) [13], local contact model (LCM) [14], weighted contact number (WCN) model [15], molecular nonlinear dynamics [16], stochastic dynamics [17] and flexibility-rigidity index (FRI) [18, 19]. In these models, biomolecular structures are usually modeled as graphs or networks, and a deterministic relationship is established between experimental B-factors and certain network properties, such as node degree, centrality, pseudo-inverse Laplacian matrix and pseudo-inverse Hessian matrixes.

Other than the above deterministic models, data-driven machine learning models are also considered in flexibility analysis [20–29], thanks to the accumulation of ever-increasing experimental data. In these learning models, biomolecular genetic, epigenetic, evolutional and structural information are extracted and used as features in machine learning models, such as support vector machine (SVM), random forest (RF), gradient boost tree (GBT) and artificial neural network (ANN). Among these learning models, an evolution-information-based learning model has been used in RNA flexibility analysis [27]. In this model, position-specific iterative basic local alignment search tool (PSI-BLAST) [30] is considered for homologous sequence identification. For each sample, a position-specific scoring matrix (PSSM) profile is calculated. The properties of the matrix are used as feature vectors and fed into various machine learning models. A Pearson correlation coefficient (PCC) value of 0.5028 between the test and predicted B-factor values has been achieved [27]. Further, more features from sequence-based information, including nucleotide acid one hot vector, predicted secondary structure, and predicted solvent accessibility, are considered in RNAbval model [29]. Combined with random forest, RNAbval can significantly improve the performance and achieve a PCC of 0.6061 [29]. Moreover, multiscale weighted colored graphs (MWCGs) based learning model is proposed to blindly predict protein B-factors [28]. These MWCGs provide a series of graph features, that characterize the intrinsic flexibility of protein structure very well. The model can be used in the blind prediction of protein B-factor with a PCC value of 0.66.

More recently, a persistent-homology (PH)-based machine learning model is proposed [28]. In this model, PH, which is a tool for data simplification and dimension reduction, is used for protein structure featurization. Different from conventional topology tools, which tend to oversimplify structural information and thus can only be used in qualitative modeling, PH manages to retain the important geometrical properties through a filtration process. Essentially, a series of simplicial complexes are generated and their topological information are characterized by homology groups [31, 32]. The “birth” and “death” of these homology generators are recorded and can be represented in either persistent diagrams (PDs) or persistent barcodes (PBs) [33]. Further, atom-specific PH and element-specific PH are used to classify the structures into different point sets with more detailed structural information [28]. Moreover, two types of matrices, one based on Euclidean distance and another on multiscale interaction, are considered. Machine learning models can achieve a PCC value up to 0.73 for a dataset of 364 proteins using the topological features extracted from their corresponding PBs [28].

Motivated by the great success of the PH-based machine learning models in protein B-factor prediction, we propose weighted-persistent-homology (WPH)-based machine learning (WPHML) models for RNA B-factor prediction. WPH incorporates physical, chemical and biological information into the topological measurements with a weight function [34, 35]. In general, different weights are assigned to *k*-simplexes with *k* starting from 0. In particular, by assigning a weight value of 0 or 1 to each point, we can naturally arrive at a local PH model and element-specific PH model [36–38]. Similarly, an interactive PH is derived by assigning weight values only to the edges between the interaction atoms [36–38]. More importantly, a weighted boundary operator can be designed to embed higher-level relations into topological invariants.

In this paper, we only consider weight values on points, i.e., atoms, to select a local region around a certain atom-of-interest, whose flexibility is to be evaluated. PH analysis is then applied to the selected atoms within the local region. Features will be generated from the corresponding PBs using a binning approach before the features are fed into learning models. To test and compare the performance of our models, the same dataset and preprocessing steps as described by Guruge et al. [27] are used. Our results show that WPH-based learning models can consistently outperform this sequence-based model in RNA B-factor prediction [27]. However, it should be noticed that higher accuracy can be achieved with more sophisticated feature engineering of sequence information [29]. A combination of features from both structure and sequence may achieve even better accuracy. Essentially, the importance of featurization and feature engineering in material, chemical and biological learning models can not be overemphasized.

The paper is organized as follows. Weighted persistent homology based featurization and the combination with different types of machine learning approaches are introduced in Section “Methodology”. In Section “Results”, we present the findings of our numerical results, including the comparison between the benchmark and our WPHML approaches and the sensitivity analysis of the model settings. The paper ends with a conclusion.

## 2 Methodology

In this section, we give a brief introduction to persistent homology and weighted persistent homology. Then, topology-based featurization is discussed in great details. After that, we briefly discuss the four main learning models that are considered.

### 2.1 Topology-based feature engineering

Data-driven sciences are widely regarded as the fourth paradigm that can fundamentally change sciences and pave the way for a new industrial revolution [39]. The past decade has witnessed a great boom of learning models in areas such as data mining, natural language processing, image analysis, animation and visualization. In contrast, the application of learning models in materials, chemistry and biology is far behind this trend.

One of the most important reasons is featurization or feature engineering [40–42]. Compared to text, image or audio data, molecular structural data from material, chemistry and biology are highly irregular and differ greatly from each other. Essentially, each molecule can have not only different numbers or types of atoms but also very different and complicated spatial connectivity. The structural complexity and high data dimensionality have significantly hampered the progress of the application of learning models in these fields.

To solve the problems, various ways of featurization have been proposed and a series of molecular descriptors (features) are generated. In general, molecular descriptors can be divided into three groups, i.e., structural measurements, physical measurements, and genetic features [40–42]. Structural measurements come from structural geometry, chemical conformation, chemical graph, structure topology, etc. Physical descriptors come from molecular formula, hydrophobicity, steric properties, and electronic properties, etc. Genetic features can be derived from gene sequences, gene expression, genetic interaction, evolution information, epigenetic information, etc.

Recently, persistent homology has been used in molecular characterization. With the unique attribute that balances geometric complexity and topological simplification, PH provides a unique structure featurization that can be naturally combined with machine learning models. PH-based learning models have been successfully used in various aspects of drug design [36–38], including protein-ligand binding affinity prediction, solubility, toxicity, and partition coefficient. More recently, PH-based learning models have been used in protein B-factor blind prediction and a remarkable high accuracy is obtained [28]. These great successes have inspired us to propose WPHML for RNA B-factor prediction. To have a better understanding of our WPHML, a brief introduction of PH and WPH is given below.

#### 2.1.1 Persistent Homology (PH)

General speaking, persistent homology can be analyzed from three aspects—graph and simplicial complex; geometric measurements and topological invariants; and a bridge between geometry and topology.

*Graph and simplicial complex*. A simplex is a generalization of the notion of a triangle or tetrahedron to arbitrary dimensions and it is the building block for the simplicical complex. A simplicial complex *K* is a finite set of simplices that satisfy two essential conditions. First, any face of a simplex in *K* is also in *K*. Second, the intersection of any two simplices in *K* is either empty or shares faces. Geometrically, a 0-simplex is a vertex, a 1-simplex is an edge, a 2-simplex is a triangle, and a 3-simplex represents a tetrahedron. Graphs and networks, composed of only vertices and edges, are special cases of simplicial complexes.

*Geometric measurements and topological invariants*. Geometry models consider geometrical information such as coordinates, distances, angles, areas, various curvatures and vector bundles. Graph models study measurements such as degree, shortest path, clique, cluster coefficient, closeness, centrality, betweenness, Cheeger constant, modularity, graph Laplacian, graph spectral, Erdős number and percolation. These geometric and graph descriptors characterize local and non-intrinsic information very well. In contrast, PH explores the intrinsic connectivity information measured by Betti number, which is a type of topological invariants that is unchanged under deformation. Geometrically, we can regard *β*_0_ as the number of isolated components; *β*_1_ the number of one-dimensional loops, circles, or tunnels, and; *β*_2_ the number of two-dimensional voids or holes.

**Fig 1:**
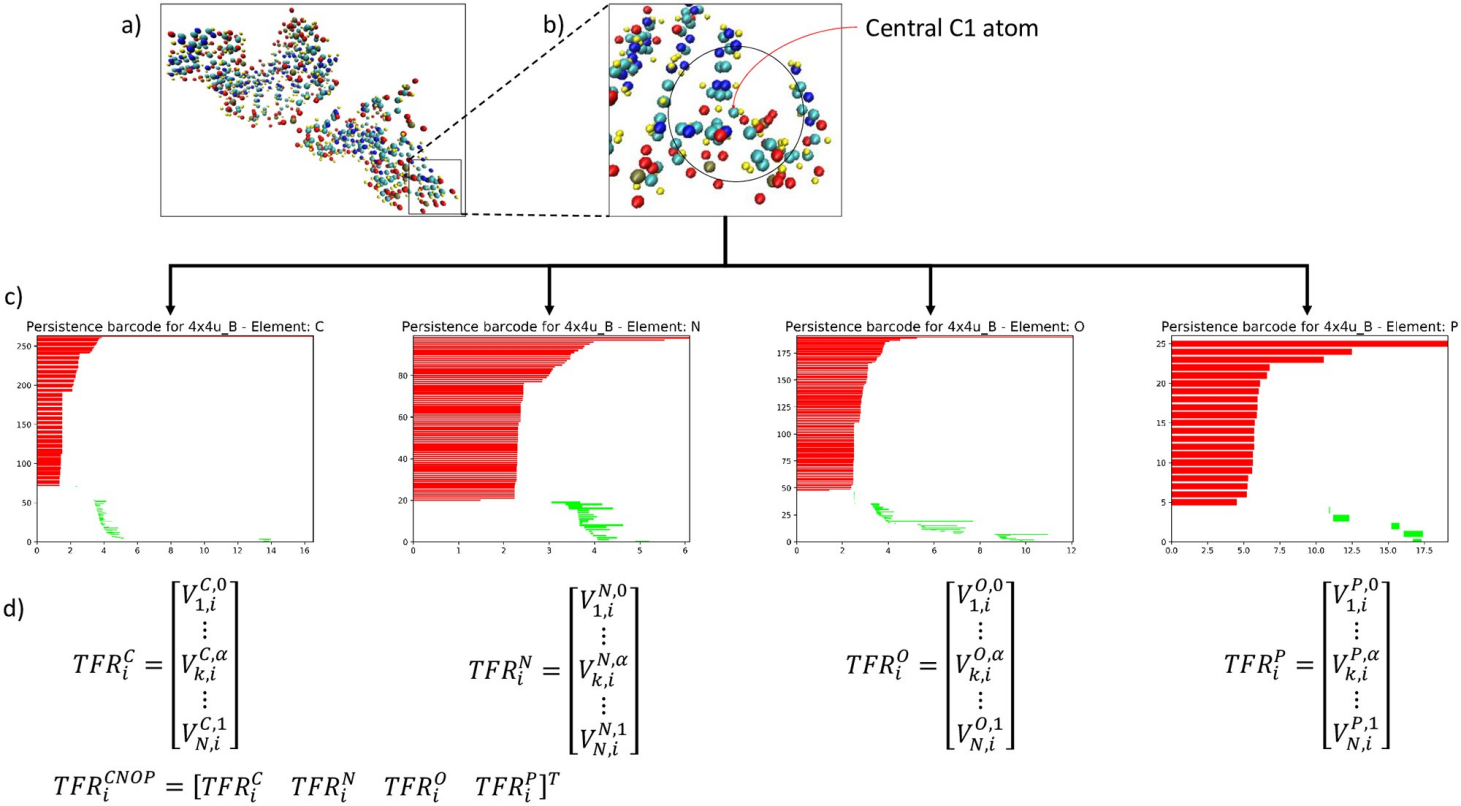
(a) Chain B of RNA 4X4U with each element (C, N, O, P and H) highlighted in a different colour. (b) In order to apply LPH, a local region and all the atoms within the local region for each central C1 atom is determined. However, only the elements of C, N, O and P are considered. (H highlighted in yellow is ignored) (c) Localized ESPH is applied and a persistent barcode is plotted for each of the four elements. (d) Binning approach is applied to each PB and the corresponding topological feature representation (TFR) of a sample *i* is obtained. The feature vector of four-element is a concatenated vector of all four TFRs. The feature vectors are then used for ML training.

*Bridge between geometry and topology*. Different from geometry and topology models, PH manages to incorporate geometrical measurements into topological invariants, thus provides a balance between geometric complexity and topological simplification. The key idea of PH is a process called filtration [31, 32]. By varying the value of a filtration parameter, a series of simplicial complexes are generated. These nested simplicial complexes encode topological information of a structure from different scales. Some topological invariants “live longer” in these simplicial complexes whereas others disappear very quickly when the filtration value increases. In this way, topological invariants can be quantified by their “lifespans” or “persisting times”, which are directly related to geometric properties. A PB can be generated from the birth, death and persistence of the topological invariants of the given dataset [33]. An example of PBs can be found in Fig 1.

#### 2.1.2 Weighted Persistent Homology (WPH)

Recently, we have systematically studied WPH models and their applications in biomolecular data analysis [34, 35, 43]. General speaking, we can define weight values, which represent physical, chemical and biological properties, on *k*-simplices, such as vertices (atom centers), edges (bonds), or higher order simplices (motif or domains). That is to say WPH can be characterized into three major categories—vertex-weighted [44–47]; edge-weighted [36, 38, 48, 49], and; general-simplex-weighted models [35, 50, 51]. These weighted values can be viewed as certain distance measurements, and PH analysis can be applied. In this way, these properties are naturally incorporated into topological measurements.

On the other hand, we can define a weighted boundary map, which can embed deeper interaction relationships into a topology. Note that to ensure the consistency of the homology definition, weight values on different simplexes need to satisfy certain constraints [35, 50, 51]. Previous PH models, including element-specific PH (ESPH) [36–38] and local persistent homology (LPH) [34] can be regarded as special cases of vertex-weighted PH. The multi-level PH, interactive PH, and electrostatic persistence [38] are essentially edge-weighted PH.

In this paper, LPH is used for RNA local structure characterization. Biologically, an RNA chain is made up of a set of nucleotides, in which the size of the set of nucleotides can range from low tens to a few thousands and above. In our LPH model, only atoms that are located within a specific Euclidean cut-off distance *E* from each C1 atom in each chain are considered. Note that only the B-factor for C1 atoms are predicted and evaluated against experimental data in the same manner as by Guruge et al. [27].

As a nucleotide constitutes of heavy atoms C, N, O, and P, in our LPH model, the localized ESPH is considered by using each of the four elements individually. That is, each element would eventually generate its own unique set of topological features representation for the specific local region. Note that for each ESPH, the central C1 atom is always included. Their topological features are drastically different from one another as shown in Fig 1(c). Indeed, ESPH is capable of retaining crucial biological information during topological simplification [52].

#### 2.1.3 Topological Features Representation (TFR)

Results from PH analysis are pairs of “birth” and “death” values for different dimensions of Betti numbers. They can be represented as PDs or PBs. However, PH results are notorious for meaningful metric definition and statistic interface. Various methods are proposed [53] including barcode statistics, tropical coordinates, binning approach, persistent image, persistent landscapes and image representations to construct topological features.

In this paper, we only consider topological features constructed using a binning approach [53]. More specifically, the filtration interval [0, *F*] is divided into *N* bins of equal size *f*. The number of barcodes which are located on each bin are then counted and used as feature vector [54, 55]. More specifically, the feature vector of a sample *i* is defined as:

$$\begin{array}{c} {\text{V}}_{i}=\left|\right|\left\{\left(b_{j},d_{j}\right)\in \text{B}\left(\alpha ,D\right)|b_{j}\leq kF/N\leq d_{j}\right\}\left|\right|\;1\leq k\leq N \end{array}$$

where ∥⋅∥ is cardinality i.e., the number of elements, of sets. Here *b*_*j*_ and *d*_*j*_ are referring to birth and death of bar *j*. **B**(*α*, *D*) is referring to the collections of barcodes with *α* referring to the selection of atoms and *D* referring to the dimension of the Betti numbers. Essentially, for each C1 atom, we have a *N* * 1 topological vector for each element and dimension of the Betti numbers.

### 2.2 Machine Learning (ML) models

After the topological features are represented as a feature vector, it can serve as input to predict B-factor values with ML algorithms. We consider four main ML models, namely regularized linear regression, tree-based methods (including random forest and extreme gradient boosting), support vector regression, and artificial neural networks. All our ML algorithms are implemented in Python (packages mentioned below refer to the packages in Python).

In the following descriptions of the ML models, we assume that we train our models with *n* data ${\left\{\left(x_{i},y_{i}\right)\right\}}_{i=1}^{n}$, where $y_{i}\in R$ is the normalized B-factor value of the *i*th sample (details of B-factor normalization will be discussed in Section Results), $x_{i}\in R^{p}$ is the structured topological feature vector of the *i*th sample, and *p* is the number of structured features. Conventionally, we denote by *ŷ* the predicted normalized B-factor value of a sample.

#### 2.2.1 Regularized linear regression

Linear regression is a straightforward yet efficient approach to model the relationship between a quantitative response and features. The incorporation of regularization can effectively address the high dimensionality setting where the number of features is larger than the sample size. The variable selection feature of the regularized linear regression makes it particularly suitable for our task as our feature vector is usually lengthy. The general formulation of regularized linear regression can be read as the following regularized minimization problem:

$$\begin{array}{c} \underset{\beta_{0}\in R,\beta \in R^{p}}{\text{min}}\sum_{i=1}^{n}{\left(y_{i}-\beta_{0}-x_{i}^{⊤}\beta \right)}^{2}+R_{\alpha }\left(\beta \right), \end{array}$$ (1)

where $R_{\alpha }\left(\cdot \right)$ is a regularization term. Once we obtain the minimizer of (1), denoted by $\left({\hat{\beta }}_{0},\hat{\beta }\right)$, we predict the B-factor value of the test data with structured feature vector *x* by $\hat{y}={\hat{\beta }}_{0}+x^{⊤}\hat{\beta }$. The specification of $R_{\alpha }$ determines the shrinkage of $\hat{\beta }$ and statistical accuracy of *ŷ* [56–60].

In our study, we consider the two typical choices of $R_{\alpha }$, namely L2-norm (${\alpha \|\beta \|}_{2}^{2}$) and L1-norm (*α*‖*β*‖_1_), where *α* is the tuning parameter that strikes the balance between efficiency and regularization. The regression problem with these two types of regularization are also known as **Ridge regression** [56] and **least absolute shrinkage and selection operator (LASSO)** [57], respectively. The advantage of LASSO over Ridge regression is its variable selection feature, which has strong interpretable power. From the LASSO results, one can tell which part of the structural information of the element is important. Both Ridge regression and LASSO are implemented with the package “scikit-learn” [61].

#### 2.2.2 Tree-based methods

Classification And Regression Tree (CART) [62] or decision tree learning is a common method used in ML. Many variations of trees have been proposed with the pruning and ensemble methods. The simple and interpretable tree-based methods have the advantage of handling high-dimensional data without further adjustments. It addresses our concern with the lengthy feature vector deduced from topological features representation. Among many candidates of tree-based methods, we consider **Random Forest (RF)** [63, 64] and **Extreme Gradient Boosting (XGBoost)** [65].

*RH*. RH is an ensemble learning method that creates a variety of decision (regression) trees independently during training, where each decision tree is constructed using a random subset of the features as split candidates. During the training of each tree, the split at each node is determined by the least-square method. In other words, for each region of each tree, we predict the B-factor value with the average of the B-factor values of the samples fallen in the region. In a regression RF, the final prediction is the average of the predicted values of all individual trees. In the implementation of ensemble trees, the number of trees, minimum number of samples at each leaf node, and the number of split candidates in each splitting, i.e., parameter *mtry*, are all tuning parameters. In our application of RF, we choose $mtry=\lceil \sqrt{p}\rceil$ following Breiman [64] and tune the other two hyperparameters. The RF is also implemented with the package “scikit-learn” [61].

*XGBoost*. Has been one of the popular ML tools used by the winning teams of many ML challenges and competitions, such as the Netflix prize [66] and various Kaggle challenges. Instead of computing the average output of all the individual trees as in a regression RF, each tree in XGBoost contributes a certain value which is added up iteratively. Such additive training or gradient boosting allows the predicted values to approach the actual values as closely as possible. In our study, we tune the number of trees and the maximum tree depth, which affects the number of leaves in the trees, while the remaining parameters are set default as defined by XGBoost. XGBoost is implemented with the package “xgboost” [65].

#### 2.2.3 Support Vector Regression (SVR)

SVR [67], as a version of the well-known support vector machine (SVM) [68] for regression, is another popular ML algorithm. The goal of an SVM model is to find a function *β*_0_ + *x*^⊤^ *β* that has at most *ϵ* deviation from the actual target values *y*_*i*_ for all the training data while trying to be as flat as possible [69]. Sometimes, the convex optimization problem is not feasible and a “soft margin” loss function is introduced [68]. The SVR model (*β*_0_, *β*) is determined by the following minimization problem:

$$\begin{array}{c} \underset{\beta_{0},\beta ,\xi ,\xi^{*}}{\text{min}}\frac{1}{2}{\left\|\beta \right\|}_{2}^{2}+C\sum_{i=1}^{n}\left(\xi_{i}+\xi_{i}^{*}\right)\;s.t.\;\{\begin{array}{ccc} y_{i}-\beta_{0}-x_{i}^{⊤}\beta_{i} & \leq & \epsilon +\xi_{i}\\ \beta_{0}+x_{i}^{⊤}\beta_{i}-y_{i} & \leq & \epsilon +\xi_{i}^{*}\\ \xi_{i},\;\xi_{i}^{*} & \geq & 0 \end{array}, \end{array}$$

where *ξ* and $\xi_{i}^{*}$ are slack variables to cope with the otherwise infeasible constraints of the optimization problem and the hyperparameter *C* determines the trade-off between the efficiency and the amount up to which deviation larger than *ϵ* is tolerable. Typically, we adopt kernel methods to transform the input features from a lower to a higher dimensional space, where a linear fit is feasible. Common choices of kernel include polynomial kernel, Gaussian kernel, and radial basis function (RBF) kernel. In our study, we have opted to use RBF kernel, i.e., $K\left(x,x^{\prime }\right)=\text{exp}(-\gamma \|x-x^{\prime }{\|}_{2}^{2})$, in our SVR model. The SVR is implemented with the package “scikit-learn” [61].

#### 2.2.4 Artificial Neural Network (ANN)

ANN has been proved to be capable of learning to recognize patterns or categorize input data after training on a set of sample data from the domain [70]. The ability to learn through training and to generalize broad categories from specific examples is the unique intelligence for ANN [71]. Different from other ML algorithms, ANN requires the user to determine the architecture of the network, such as the number of hidden layers, the number of nodes, and the specification of activation function in each layer. The hidden layers in ANN architecture allow the ANN to deal with nonlinear and complex problems more robustly and therefore can operate on more interesting problems [72]. The number of hidden layers enables a trade-off between smoothness and closeness of fit [73]. The number of nodes within a hidden layer determines the trade-off between training time and training accuracy. The weights of each layer are optimized via the use of a learning algorithm called “backpropagation” [74]. Since the ANN will involve the learning of a vast amount of weights, from the statistical perspective, the overfitting problem arises. We adopt a recently proposed regularization technique called “dropout” [75], which is empirically proven magical. This approach also addresses the curse of dimensionality due to the lengthy topological feature vector in our study.

In our study, the number of hidden layers, number of nodes in each hidden layer, and the number of epochs are treated as hyperparameters. The hidden and output activation functions are set as sigmoid and leaky ReLU functions respectively. The dropout rate is set to 20% and the remaining hyperparameters are set to default values as defined by the package. ANN is implemented with the package “keras” [76].

### 2.3 Model setting

#### RNA dataset

We consider the same RNA dataset and data preprocessing by Guruge et al. [27]. The chains are randomly split in the same manner with 75% of the chains go into a training set and 25% go into the test set. The B-factor of each nucleotide is represented by its C1 atom.

#### B-factor normalization and outlier detection

The values of B-factors may differ significantly from chain to chain due to reasons such as a relatively small number of residues in a protein chain or differences in refinement methods used [77]. Thus, the B-factors of each chain are normalized to have zero mean and unit variance [27]. The range of normalized B-factor falls approximately between -3.00 and 4.00. Further, before the raw B-factors are normalized, values of outliers are first detected and removed using a median-based approach [78]. This is to eliminate raw B-factor values that are located on the extreme ends of the distribution.

#### Hyperparameter setting

In our dataset, cut-off distance *E*, *F/E* ratio, and bin size *f* are the hyperparameters to be optimized. We chose the value of *E* to be in the range from 10 Å to 45 Å with a stepsize of 5 Å, i.e., *E* = {10 Å, 15 Å, 20 Å, 25 Å, 30 Å, 35 Å, 40 Å, 45 Å}. The filtration interval *F* is defined such that the ratio of *F/E* is between 0.5 to 1.0 with a stepsize of 0.1, i.e., *F/E* = {0.5, 0.6, 0.7, 0.8, 0.9, 1.0}. Bin size *f* is chosen to be in the range from 0.15 Å to 1.50 Å, i.e., *f* = {0.15 Å, 0.50 Å, 1.00 Å, 1.50 Å}. A total of 32,823 PBs are generated based on the Vietoris-Rips complex for each combination of element type, *E* and *F/E* ratio. Both “GUDHI” [79] and “Dionysus” [80] packages are used.

To determine the optimal hyperparameter values for each ML model, we conduct a five-fold cross validation (CV) using the training set. Specifically, the training set is randomly divided into five folds with a similar number of chains. In each fold, for each combination of the hyperparameters, we find the predicted B-factor values for the left-out training set with the ML model trained by the remaining training set. The optimal hyperparameter set maximizes the out-of-sample PCC between the predicted and actual values across all folds. The optimal hyperparameter values for each ML model can be found in Table 1.

**Table 1:** Optimal hyperparameter values for each ML model. ESPH—*χ* and ESPH—CNOP refer to the optimal hyperparameter values of the ML model under single-element and four-element-combined dataset respectively.

ML model	Hyperparameters	ESPH—*χ*	ESPH—CNOP
Ridge	Alpha	500	500
LASSO	Alpha	0.01	1
RF	No of trees	500	2000
	No of min samples at nodes	5	5
XGBoost	No of trees	50	50
	Tree depth	3	3
SVM	Kernel	RBF	RBF
	Gamma	0.01	0.001
	C	0.1	0.1
ANN	No of hidden layers	4	3
	No of nodes per hidden layer	68	900
	Activation type for hidden layer	Sigmoid	Sigmoid
	Dropout rate	20%	20%
	No of epochs	15	10

Once the hyperparameter values of the dataset and models have been optimized, the trained models are evaluated using a test set that was non-overlapping with the training set. The PCC between the predicted and actual normalized B-factor values in the test set is calculated for each model

$$\begin{array}{c} PCC\left(y_{i},{\hat{y}}_{i}\right)=\frac{\sum_{i=1}^{n}\left(y_{i}-\bar{y}\right)\left({\hat{y}}_{i}-\bar{\hat{y}}\right)}{\sqrt{\sum_{i=1}^{n}{\left(y_{i}-\bar{y}\right)}^{2}}\sqrt{\sum_{i=1}^{n}{\left({\hat{y}}_{i}-\bar{\hat{y}}\right)}^{2}}}, \end{array}$$

where ${\hat{y}}_{i}$ is the predicted *i*-th B-factor value, $\bar{y}=\frac{1}{n}\sum_{i=1}^{n}y_{i}$ and $\bar{\hat{y}}=\frac{1}{n}\sum_{i=1}^{n}{\hat{y}}_{i}$.

## 3 Results

In this section, we demonstrate the performance of our WPHML model. Table 2 shows the best performance achieved by each ML model on test set. The best performance reported by Guruge et al. [27] is used as a benchmark performance (benchmark PCC = 0.5028). The conditions in which the best test performances are obtained can be found in Table 3. In RNAbval model, sequence-based information, including PSSM, nucleotide acid one hot vector, predicted secondary structure, and predicted solvent accessibility, are considered [29]. By the use of extensive sequence-based features, they can achieve better result.

**Table 2:** Best test set performance for each ML model using the optimal hyperparameter values for dataset and ML model. PSSM stands for Position Specific Scoring Matrix, which is the benchmark performance by Guruge et al. [27].

Feature type	ML model	Test set PCC	Improvement (%)
PSSM	SVM (RBF)	0.5028	
ESPH—O	Ridge	0.4283	-14.8%
ESPH—O	LASSO	0.4667	-7.2%
ESPH—P	**RF**	**0.5788**	**15.1%**
ESPH—P	XGBoost	0.5748	14.3%
ESPH—P	SVM (RBF)	0.5520	9.8%
ESPH—P	ANN	0.5732	14.0%
ESPH—CNOP	Ridge	0.4849	-3.6%
ESPH—CNOP	LASSO	0.4157	-17.3%
ESPH—CNOP	**RF**	**0.5822**	**15.8%**
ESPH—CNOP	XGBoost	0.5657	12.5%
ESPH—CNOP	SVM (RBF)	0.5560	10.6%
ESPH—CNOP	ANN	0.5609	11.6%
PSSM, etc [29]	**RF**	**0.6061**	**20.5%**

**Table 3:** Best test performance conditions.

Element type(s)	ML model	Cut-off (Å)	F/E ratio	Bin size (Å)	PCC
ESPH—O	Ridge	25	0.7	1.50	0.4283
ESPH—O	LASSO	25	0.5	0.50	0.4667
ESPH—P	RF	45	0.7	0.15	0.5788
ESPH—P	XGBoost	45	0.9	1.00	0.5748
ESPH—P	SVM (RBF)	40	0.5	1.00	0.5520
ESPH—P	ANN	45	1.0	1.00	0.5732
ESPH—CNOP	Ridge	35	0.6	0.50	0.4849
ESPH—CNOP	LASSO	25	0.5	0.50	0.4157
ESPH—CNOP	RF	40	0.5	0.15	0.5822
ESPH—CNOP	XGBoost	45	0.7	0.15	0.5657
ESPH—CNOP	SVM (RBF)	35	0.5	0.15	0.5560
ESPH—CNOP	ANN	45	0.5	0.15	0.5609

For both single-element and four-element-combined models, it can be seen that WPHML models are able to consistently outperform the evolution-based method (PSSM) by at least approximately 10% with only the exception of linear regression models (Ridge and LASSO). Among all the models, RF achieves the best result with PCC = 0.5788 (15.1% improvement). Moreover, the performance of the RF model further improves to 0.5822 when the topological features for all four elements were used, which is about 15.8% improvement.

The comparison between the results from single-element and four-element-combined models shows that generally there is no significant improvement. In fact, SVM improves only slightly (approximately 0.8%), while XGBoost and ANN models even show some small reduction of accuracy (1.8% and 2.4% respectively). The results seem to be different from previous studies that concluded that element-specific models always deliver better results [28, 36–38]. Note that previous models are based on protein structures.

Comparably speaking, RNA structures are more regular and relatively simple. Similar topological features may be embedded in different types of element models. In this way, the additional features do not incorporate new information, instead they will contribute more noises, which causes the drop in performances. Noted that the best test performance of all the models except linear regression using a single element are all based on element P.

### Effect of Euclidean cut-off distance

**Fig 2:**
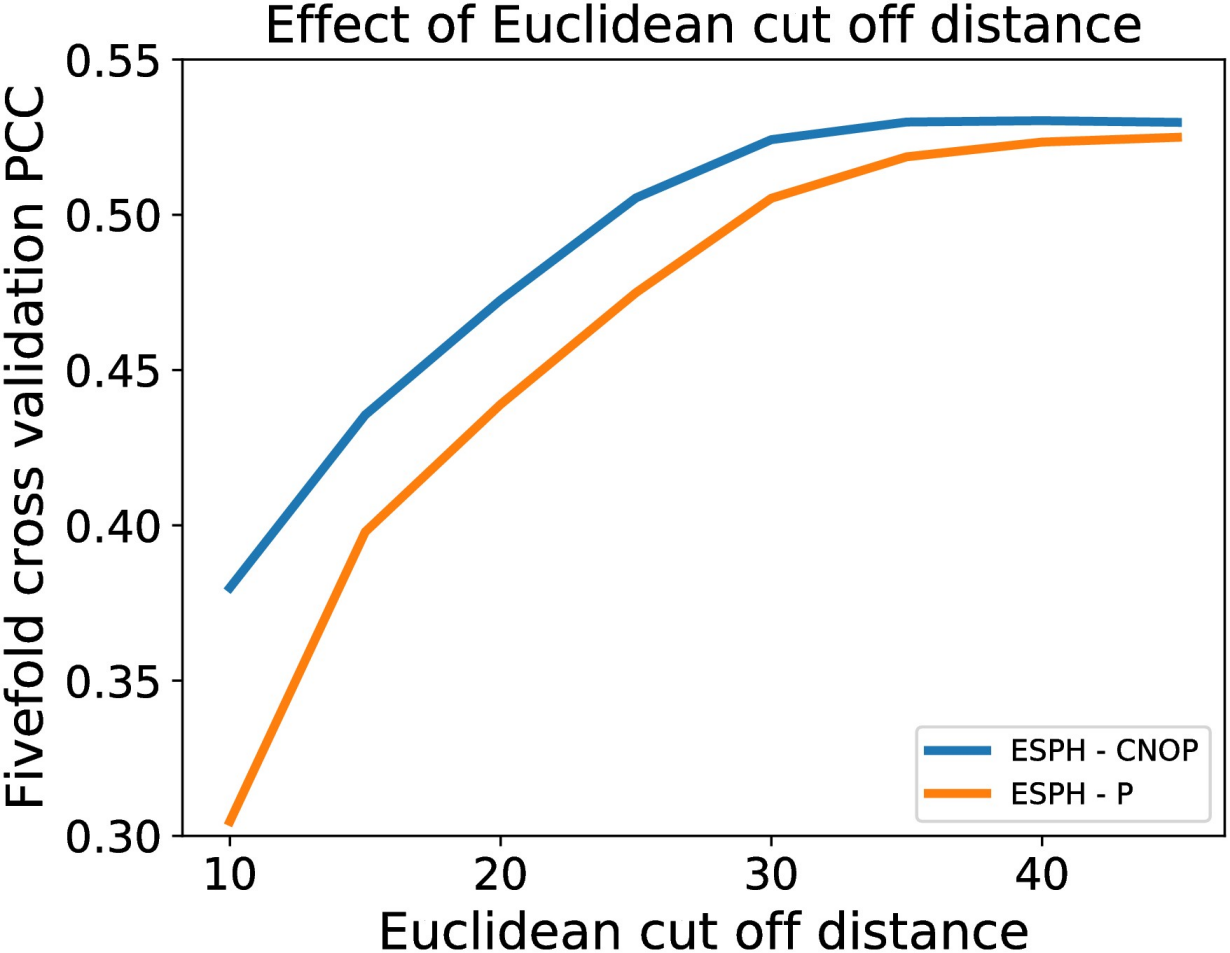
Effect of Euclidean cut-off distance on RF using the topological features from element P and all four elements with a fixed F/E ratio of 1.0 and bin size of 0.15Å.

Fig 2 shows the effect of cut-off distance. It can be seen that the PCCs of the fivefold cross validation using the topological features from both element P and all four elements gradually improve and eventually plateaus off at approximately 35 Å. Note that 35 Å is larger than the generally used cut-off distance in the Gaussian network model, anisotropic network model, and other graph-based models, which are usually around 8 Å to 20 Å.

One of the reasons that larger cut-off distance delivers good results is that our predicted PCC values are predominantly determined by the several larger-sized RNAs. From S1 Table, it can be seen that there is a wide range of chain PCC distribution in the test set, which ranges from -0.50 to 0.80 although our RF model has a fairly good PCC of 0.5822. Moreover, approximately 70% of the test data points come from 4 out of the 34 chains, of which these 4 chains have a chain PCC higher than the overall PCC achieved by the RF model. With that said, the performance of the test set is heavily based on these 4 chains. As long as the predictions on these 70% data points continue to improve, the overall performance of the model would continue to improve although there may be a reduction in performance on the remaining 30% of data points. This indicates that the evaluation method [27, 28] may have certain limitations. However, for a fair comparison, we still use it in the current paper.

### Effect of F/E ratio

**Fig 3:**
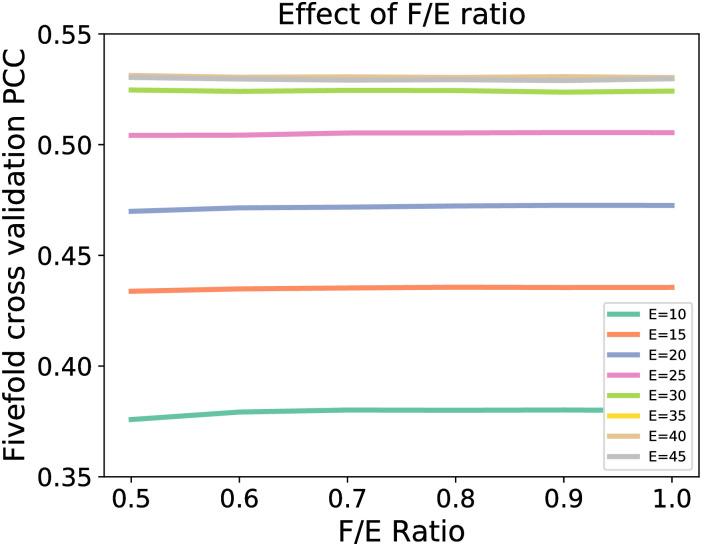
Effect of F/E ratio on RF using the topological features from all four elements and bin size of 0.15Å.

Fig 3 shows the effect of F/E ratio on the fivefold cross validation performance. At a low cut-off distance, the improvement in the fivefold cross validation performance improves more significantly when F/E ratio increases from 0.5 to 0.7. Beyond 0.7, the improvement in performance is very minimal. However, at a large cut-off distance, the performance is rather consistent from 0.5 to 1.0. This shows that the F/E ratio is not a significant hyperparameter to generate the dataset and it is more than sufficient to use an F/E ratio of 0.5 so as to minimize the number of unnecessary features generated especially as a large cut-off distance is required as discussed previously.

### Effect of bin size

**Fig 4:**
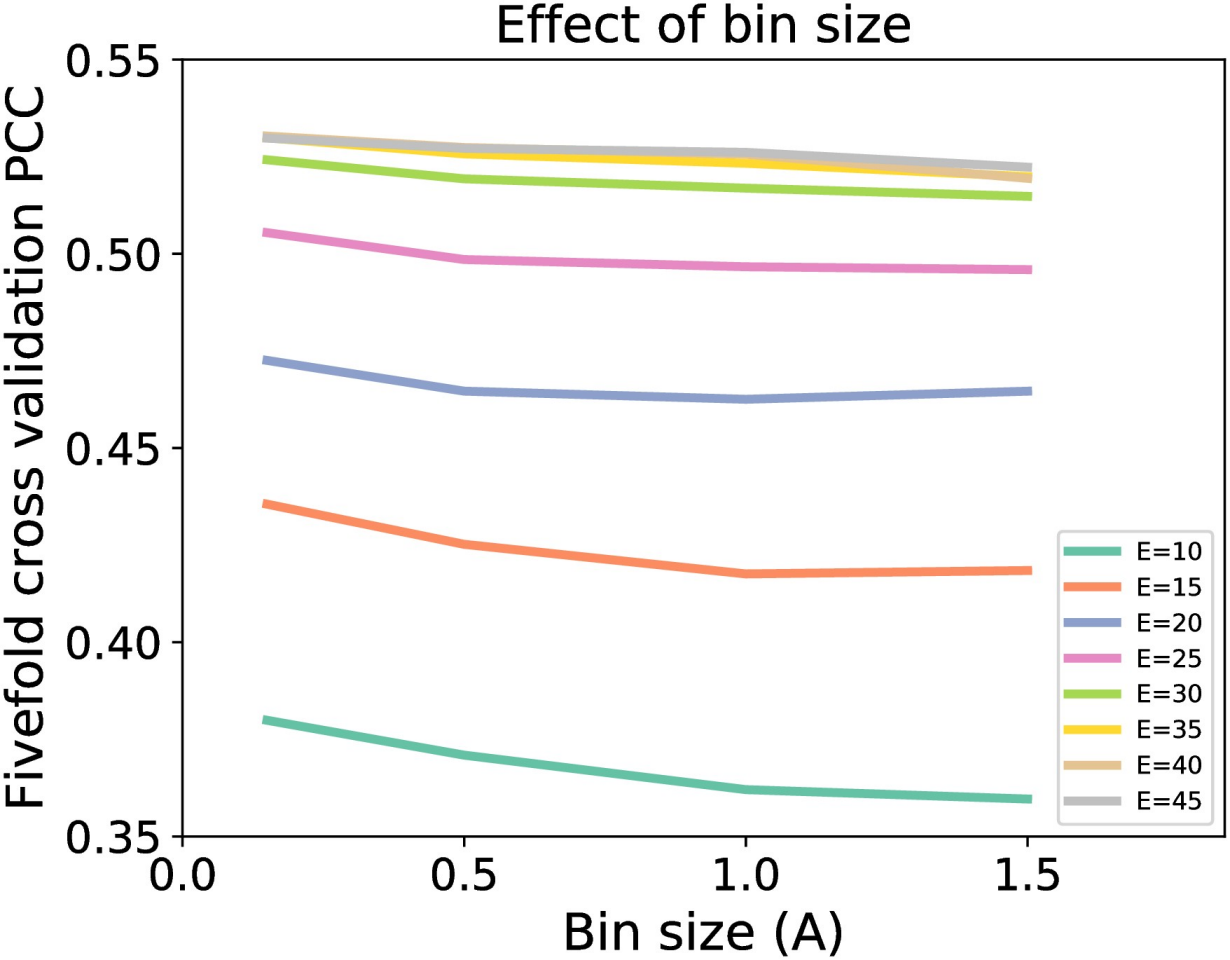
Effect of bin size on RF performance with a fixed F/E ratio of 1.0.

Fig 4 shows the changes in five-fold CV performance with respect to bin size. As the bin size decreases from 1.5 Å to 0.15 Å, the performance improves for all Euclidean cut-off distance. This indicates that with a smaller bin size, the finer details of topological features are detected especially topological invariants that exist for a very short moment. The geometric information, embedded in the topological invariants, are key to the success of WPHML models.

## 4 Conclusion

In this paper, we propose the weighted-persistent-homology-based machine learning (WPHML) models and use them in the RNA B-factor prediction. We found that our WPHML models can consistently deliver a better performance than the evolution-based learning models. In particular, local persistent homology and element-specific persistent homology are considered for topological feature generation. These topological-feature-based random forest models can deliver a PCC up to 0.5822, which is 15.8% increase as compared to the performance of the previous model. Our WPHML models are suitable for any biomolecular-structure-based data analysis. Note that more sophisticated feature engineering of sequence-based information can further improve the accuracy to 0.61 [29]. This again demonstrates the great importance of featurization for material, chemical and biological learning models.

## Supporting information

S1 Table PCC of each RNA chain in training set achieved by the best optimal RF. (PDF)

S2 Table PCC of each RNA chain in test set achieved by the best optimal RF. (PDF)
