# Supplementary material for: Weighted-persistent-homology-based machine learning for RNA flexibility analysis
Source: PLoS One. 2020 Aug 21;15(8):e0237747. doi: 10.1371/journal.pone.0237747 (PMC7446851; doi:10.1371/journal.pone.0237747)
Supplement: S1 Table — (PDF) [file pone.0237747.s001.pdf]

S1 Table : PCC of each RNA chain in training set achieved by the best optimal RF.

Begin of Table I				
Chain	End-to-end distance	Chain size	Percentage of training set	Chain PCC
1asy_R	74.52	67	0.2%	0.9079
1b23_R	87.30	66	0.2%	0.9101
1c0a_B	74.72	68	0.2%	0.9308
1ddy_A	56.63	35	0.1%	0.8727
1f1t_A	53.37	32	0.1%	0.8237
1ffk_9	110.70	121	0.4%	0.8976
1ffy_T	81.21	74	0.3%	0.8686
1g1x_D	83.59	39	0.1%	0.8760
1g59_B	78.86	75	0.3%	0.9121
1gax_C	85.39	75	0.3%	0.9375
1h3e_B	74.16	80	0.3%	0.8953
1h4q_T	72.18	64	0.2%	0.9117
1i6u_C	58.87	37	0.1%	0.8654
1j1u_B	81.41	73	0.3%	0.8438
1kxk_A	95.33	69	0.2%	0.9157
1l9a_B	113.22	124	0.4%	0.9527
1m5k_B	102.67	91	0.3%	0.9526
1qf6_B	73.34	69	0.2%	0.9550
1s03_A	68.57	47	0.2%	0.9036
1ser_T	66.79	62	0.2%	0.8586
1sj3_R	79.10	72	0.3%	0.9228
1ttt_D	83.16	62	0.2%	0.8942
1u9s_A	115.07	155	0.6%	0.9097
1vfg_C	47.42	31	0.1%	0.7358
1vy5_AX	87.82	72	0.3%	0.8837
1xjr_A	65.92	46	0.2%	0.8916
1y26_X	70.52	70	0.2%	0.9126
1y27_X	68.09	67	0.2%	0.8980
1yfg_A	87.14	64	0.2%	0.9810
1yls_B	57.26	33	0.1%	0.7753
1zho_B	49.06	38	0.1%	0.7856
2azx_C	78.27	72	0.3%	0.9117
2cky_A	62.84	77	0.3%	0.8479
2csx_C	77.30	75	0.3%	0.9007
2dlc_Y	79.89	63	0.2%	0.7992
2du3_D	77.64	71	0.3%	0.8841
2fk6_R	58.00	51	0.2%	0.7857
2gcs_B	101.87	122	0.4%	0.9190
2gis_A	70.62	94	0.3%	0.9105
2hoj_A	69.93	77	0.3%	0.8668
2hvy_E	82.76	61	0.2%	0.7958
2nue_C	71.75	45	0.2%	0.8690
2nz4_P	123.18	140	0.5%	0.9433
2oeu_A	63.02	42	0.1%	0.8001
2qbz_X	105.64	153	0.5%	0.9729
2qex_0	223.35	2740	9.8%	0.9631

Continuation of Table I				
Chain	End-to-end distance	Chain size	Percentage of test dataset	Chain PCC
2qus_A	87.61	68	0.2%	0.8835
2qwy_A	64.52	52	0.2%	0.8904
2r8s_R	116.33	158	0.6%	0.9177
2vpl_B	64.55	48	0.2%	0.9015
2xdb_G	63.43	34	0.1%	0.8177
2zh2_B	61.40	34	0.1%	0.7996
2zjr_X	219.60	2685	9.6%	0.9635
2zjr_Y	105.12	121	0.4%	0.8933
2zue_B	81.15	75	0.3%	0.8856
2zzm_B	75.64	84	0.3%	0.8865
2zzn_C	74.81	71	0.3%	0.9379
3adb_C	86.47	92	0.3%	0.9345
3akz_F	83.85	74	0.3%	0.8983
3am1_B	81.67	81	0.3%	0.8846
3amt_B	84.81	78	0.3%	0.9104
3cc2_0	222.12	2740	9.8%	0.9641
3cul_C	81.86	91	0.3%	0.9310
3dig_X	108.56	173	0.6%	0.9091
3e5c_A	63.14	52	0.2%	0.9475
3egz_B	78.94	65	0.2%	0.9070
3eph_E	76.21	69	0.2%	0.9306
3f2q_X	77.26	107	0.4%	0.8954
3fu2_A	42.22	31	0.1%	0.8248
3g78_A	121.02	388	1.4%	0.8987
3gca_A	46.73	32	0.1%	0.7200
3gs5_C	61.00	35	0.1%	0.7956
3hhn_C	85.52	135	0.5%	0.8988
3hl2_E	82.55	82	0.3%	0.9323
3iab_R	75.56	46	0.2%	0.8980
3irw_R	76.11	90	0.3%	0.8968
3kfu_K	87.73	71	0.3%	0.9002
3ndb_M	130.49	135	0.5%	0.9388
3nmu_D	62.64	34	0.1%	0.9603
3npn_A	65.37	50	0.2%	0.8616
3ouy_C	62.07	35	0.1%	0.8929
4n0t_B	70.02	65	0.2%	0.9465
4o26_E	63.51	47	0.2%	0.8791
4oji_A	56.33	51	0.2%	0.7844
4oog_D	60.78	34	0.1%	0.8030
4p5j_A	75.21	83	0.3%	0.9217
4p95_A	102.31	188	0.7%	0.8799
4pdb_I	59.44	38	0.1%	0.8718
4pkd_V	85.57	54	0.2%	0.9054
4pqv_A	62.13	68	0.2%	0.8610
4pr6_B	81.05	71	0.3%	0.8924
4qlm_A	78.00	108	0.4%	0.8728
4rdx_C	79.59	76	0.3%	0.8612
4rge_A	61.66	54	0.2%	0.8438
4ts0_X	92.27	42	0.1%	0.9281
4u3m_1	250.52	3149	11.2%	0.9667
4u3m_2	265.88	1750	6.2%	0.9619
4u3m_3	111.81	121	0.4%	0.9540
4u3m_4	162.52	158	0.6%	0.9299
4u7u_L	129.24	60	0.2%	0.9674
4v51_BA	218.17	2771	9.9%	0.9266
4v67_AA	243.28	1503	5.4%	0.9346
4v67_BB	106.55	118	0.4%	0.8578
4v8b_AB	91.51	87	0.3%	0.9551
4v8d_AB	90.47	84	0.3%	0.9329
4v90_AV	88.85	75	0.3%	0.9183

Continuation of Table I				
Chain	End-to-end distance	Chain size	Percentage of test dataset	Chain PCC
4v9o_AA	235.27	2854	10.2%	0.9562
4v9o_AB	105.13	118	0.4%	0.9214
End of Table I				
