# Supplementary material for: Weighted-persistent-homology-based machine learning for RNA flexibility analysis
Source: PLoS One. 2020 Aug 21;15(8):e0237747. doi: 10.1371/journal.pone.0237747 (PMC7446851; doi:10.1371/journal.pone.0237747)
Supplement: S2 Table — (PDF) [file pone.0237747.s002.pdf]

S2 Table: PCC of each RNA chain in test set achieved by the best optimal RF.

Begin of Table II				
Chain	End-to-end distance	Chain size	Percentage of test dataset	Chain PCC
1dk1_B	74.59	56	0.9%	0.0239
1dul_B	70.38	47	0.7%	-0.4194
1kh6_A	56.33	42	0.7%	-0.0116
2bte_B	82.27	78	1.2%	0.3136
2oiu_P	72.68	71	1.1%	-0.1482
2ozb_C	53.11	32	0.5%	-0.2372
3hjw_D	88.36	57	0.9%	0.2195
3owi_A	98.22	86	1.3%	0.4812
3p22_A	61.22	39	0.6%	-0.4328
3q3z_V	78.02	74	1.1%	0.5131
3rw6_H	58.09	60	0.9%	0.1577
3sd3_A	81.21	89	1.4%	0.1356
3ski_A	64.25	66	1.0%	0.3185
3suh_X	89.83	100	1.6%	0.1145
3v7e_C	93.40	125	1.9%	0.1623
3vjr_B	58.70	36	0.6%	-0.3115
3vrs_A	61.23	51	0.8%	0.5918
3zgz_B	78.94	81	1.3%	0.2161
4ato_G	60.24	32	0.5%	-0.1138
4c7o_E	67.90	48	0.7%	-0.1835
4fnj_A	59.37	34	0.5%	0.2358
4frg_B	76.24	84	1.3%	-0.2167
4jf2_A	78.06	76	1.2%	0.0851
4jrc_A	68.34	56	0.9%	-0.1906
4k27_U	81.07	55	0.9%	0.7721
4kr6_C	58.05	38	0.6%	0.2246
4kzd_R	102.80	83	1.3%	-0.1545
4l81_A	69.99	96	1.5%	0.2590
4lnt_RA	221.56	2881	44.8%	0.7864
4m4o_B	68.42	59	0.9%	-0.4869
4v9o_BA	238.96	1533	23.8%	0.6667
4wfl_A	89.57	105	1.6%	-0.0519
4x4p_B	58.08	36	0.6%	0.0530
4x4u_B	59.39	31	0.5%	0.1802
End of Table II				
